# Study Protocol for Preventing Early-Onset Pneumonia in Young Children Through Maternal Immunisation: A Multi-Centre Randomised Controlled Trial (PneuMatters)

**DOI:** 10.3389/fped.2021.781168

**Published:** 2022-01-17

**Authors:** Anne B. Chang, Maree Toombs, Mark D. Chatfield, Remai Mitchell, Siew M. Fong, Michael J. Binks, Heidi Smith-Vaughan, Susan J. Pizzutto, Karin Lust, Peter S. Morris, Julie M. Marchant, Stephanie T. Yerkovich, Hannah O'Farrell, Paul J. Torzillo, Carolyn Maclennan, David Simon, Holger W. Unger, Hasthika Ellepola, Jens Odendahl, Helen S. Marshall, Geeta K. Swamy, Keith Grimwood

**Affiliations:** ^1^Child Health Division and NHMRC Centre for Research Excellence in Paediatric Bronchiectasis (AusBREATHE), Menzies School of Health Research, Charles Darwin University, Casuarina, NT, Australia; ^2^Australian Centre for Health Services Innovation, Queensland University of Technology, Brisbane, QLD, Australia; ^3^Department of Respiratory Medicine, Queensland Children's Hospital, Brisbane, QLD, Australia; ^4^Faculty of Medicine, The University of Queensland, St. Lucia, QLD, Australia; ^5^Division of Paediatric Infectious Diseases, Hospital Likas, Kota Kinabalu, Malaysia; ^6^Women's and Newborn Services, Royal Brisbane and Women's Hospital, Herston, QLD, Australia; ^7^Central Clinical School, University of Sydney, Sydney, NSW, Australia; ^8^Prince Alfred Hospital, Sydney, NSW, Australia; ^9^Department of Obstetrics and Gynaecology, Royal Darwin Hospital, Tiwi, NT, Australia; ^10^Department of Obstetrics and Gynaecology, Logan Hospital, Meadowbrook, QLD, Australia; ^11^Vaccinology and Immunology Research Trials Unit, Women's and Children's Health Network, Adelaide Medical School, Robinson Research Institute, The University of Adelaide, Adelaide, SA, Australia; ^12^Division of Maternal-Fetal Medicine, Department of Obstetrics and Gynecology, Duke University Health System, Durham, NC, United States; ^13^Department of Infectious Disease and Paediatrics, Gold Coast Health, Southport, QLD, Australia; ^14^School of Medicine and Dentistry, Menzies Health Institute Queensland, Griffith University, Southport, QLD, Australia

**Keywords:** maternal immunisation, pneumonia, children, randomised controlled trial, protocol

## Abstract

**Background:** Preventing and/or reducing acute lower respiratory infections (ALRIs) in young children will lead to substantial short and long-term clinical benefits. While immunisation with pneumococcal conjugate vaccines (PCV) reduces paediatric ALRIs, its efficacy for reducing infant ALRIs following maternal immunisation has not been studied. Compared to other PCVs, the 10-valent pneumococcal-*Haemophilus influenzae* Protein D conjugate vaccine (PHiD-CV) is unique as it includes target antigens from two common lower airway pathogens, pneumococcal capsular polysaccharides and protein D, which is a conserved *H. influenzae* outer membrane lipoprotein.

**Aims:** The primary aim of this randomised controlled trial (RCT) is to determine whether vaccinating pregnant women with PHiD-CV (compared to controls) reduces ALRIs in their infants' first year of life. Our secondary aims are to evaluate the impact of maternal PHiD-CV vaccination on different ALRI definitions and, in a subgroup, the infants' nasopharyngeal carriage of pneumococci and *H. influenzae*, and their immune responses to pneumococcal vaccine type serotypes and protein D.

**Methods:** We are undertaking a parallel, multicentre, superiority RCT (1:1 allocation) at four sites across two countries (Australia, Malaysia). Healthy pregnant Australian First Nation or Malaysian women aged 17–40 years with singleton pregnancies between 27^+6^ and 34^+6^ weeks gestation are randomly assigned to receive either a single dose of PHiD-CV or usual care. Treatment allocation is concealed. Study outcome assessors are blinded to treatment arms. Our primary outcome is the rate of medically attended ALRIs by 12-months of age. Blood and nasopharyngeal swabs are collected from infants at birth, and at ages 6- and 12-months (in a subset). Our planned sample size (*n* = 292) provides 88% power (includes 10% anticipated loss to follow-up).

**Discussion:** Results from this RCT potentially leads to prevention of early and recurrent ALRIs and thus preservation of lung health during the infant's vulnerable period when lung growth is maximum. The multicentre nature of our study increases the generalisability of its future findings and is complemented by assessing the microbiological and immunological outcomes in a subset of infants.

**Clinical Trial Registration:** https://www.anzctr.org.au/Trial/Registration/TrialReview.aspx?id=374381, identifier: ACTRN12618000150246.

## Introduction

The global burden of disease study estimated that acute lower respiratory infections (ALRIs) caused 2.74 million [95% uncertainty interval (UI) 2.50–2.86 million] deaths in 2015 with much of the burden borne by children aged <5-years [704,000 deaths (95% UI 651,000–763,000)] living in low-middle income countries (LMICs) (1). The global morbidity of ALRIs is also high (~120 million episodes in children aged <5-years) in both LMICs and high-income countries (2). In the United States (US), ALRIs are the leading cause of hospitalisation during infancy (2). Furthermore, the highest published rates of hospitalised ALRI and radiographically-confirmed pneumonia in children aged <5-years are in First Nations people living in high-income countries. In the Northern Territory of Australia, where childhood vaccine uptake is high, the incidence rate of hospitalised ALRI in First Nations children in the first year of life is 427 episodes per 1000 child-years [95% confidence interval (CI) 416–437], while in Central Australia incidence rates for radiographically-confirmed pneumonia are 27 per 1,000 child-years (95% CI 25–28) in those aged <5-years and 78.4 per 1,000 child-years (95% CI 68–90) in those <1-year of age (3).

Whilst the global mortality of childhood ALRIs has declined (1), morbidity and long-term outcomes of ALRIs remain an important public health issue. ALRIs risk injuring the developing lung, which may lead to subsequent impaired lung growth and function (4, 5) and predispose children to chronic lung disease (6). Our previous international cohort (7) study found that early-onset ALRI requiring hospitalisation (first episode occurred at median age of 3.7-months) was an independent risk factor for bronchiectasis. Similarly, First Nations children hospitalised with ALRI previously were 15-times more likely than controls (First Nations children hospitalised for other reasons) to develop bronchiectasis [odds ratio (OR) 15, 95% CI 4–53] and the risk increased further in those with recurrent ALRI episodes (8).

It is being appreciated increasingly that ALRIs early in life are associated with lung function deficits in older children and adults and also with chronic respiratory illnesses (4, 5), especially chronic obstructive pulmonary disease (COPD). ALRIs in the first 2-years of life are significantly associated with COPD (9) and non-smoking related COPD is now recognised as a major global health problem (9, 10). Both bronchiectasis and COPD are common among disadvantaged “at-risk populations” encountered in LMICs (11) and amongst First Nations populations (12). Thus, intervention strategies that reduce infant ALRIs, especially in “at-risk populations” will likely have both immediate and large long-term benefits.

One strategy to reduce infant ALRIs is maternal immunisation. Physiological and immunological changes during pregnancy result in an increased infection risk for both the mother and foetus (13). Maternal immunisation could protect the mother against infection and its adverse consequences for the foetus and also provide passive protection against infection in the first months of life for the newborn infant (14). Transfer of maternal immunoglobulin (Ig) G starts from the second trimester (15) but the most active transplacental transport of maternal immunoglobulin (Ig) G occurs after 32-weeks gestation (14). Hence immunising pregnant woman between 28 and 32-weeks (a robust IgG response takes ~4-weeks) is the ideal timing to maximise transplacental transfer of vaccine-specific IgG for passive infant protection prior to priming from vaccines initiated in early infancy (14).

Maternal immunisation is a rapidly developing field offering tremendous large-scale benefits for mothers and infants (16–18). Recognising the potential of maternal immunisation as a prevention strategy, the US National Institute of Health sponsored a series of meetings toward its development (16). The Global Alignment of Immunisation Safety Assessment in pregnancy (GAIA) consortium was also formed and published a series of papers to standardise methodology and definitions (19). Current US (18) and Australian (20) guidelines recommend both the combination diphtheria-tetanus-acellular pertussis (dTaP) and seasonal influenza vaccines for all pregnant women as there is proven efficacy of their positive impact. Although pneumococcal vaccines during pregnancy are recommended when selected risk factors are present in the mother, there are insufficient data to offer routine pneumococcal vaccination for all pregnant women (18, 20). Neonatal vaccination is an alternative strategy, but is inferior to maternal immunisation as newborn infants have limited Th1 responses to many vaccine antigens (14, 21). Thus, unlike maternal immunisation, neonatal immunisation with pneumococcal vaccines does not reliably provide sufficient protection during the first weeks of life (22), although reassuringly does not appear to induce immune tolerance.

Many pathogens cause ALRI in young children. Globally, *Streptococcus pneumoniae* (pneumococci) is reported to be the largest contributor to deaths annually from ALRIs in all age groups [55.4% or 1.52 million (95% UI 858,000–2.18 million)], including children <5-years of age [55.8% or 393,000 (95%UI 228,000–532,000)] (1). Also, pneumococci remain the most common cause of community-acquired pneumonia in adults (23). Unsurprisingly, pneumococcal infection was one of the most common ALRIs in high-income countries before pneumococcal conjugate vaccines (PCV) were introduced (24). As Australian First Nations children and adults have an increased risk of ALRI and pneumococcal infection, they have a different immunisation schedule to other Australians (20) and had PCVs several years earlier than other community groups (3, 25). Currently, Northern Territory First Nations children receive a 13-valent PCV (PCV-13 or Prevenar-13) at 2, 4 and 6-months of age as their primary course and a booster dose at age 12-months (3 + 1 schedule), while all adults aged ≥50-years receive PCV-13 followed by two doses of the 23-valent pneumococcal polysaccharide vaccine (PPV-23) 5-years apart (20). In contrast, the national immunisation program schedule for other Australians is two doses of PCV-13 at 2 and 4-months of age with a booster at 12-months (2 + 1 schedule) and a single dose of PCV-13 at age 70-years (20). Thus, the potential beneficial effects of protecting the infant against pneumococcal infections align with the data supporting a maternal immunisation strategy to reduce ALRIs, especially for First Nations infants.

There are several licenced pneumococcal vaccines, the old PPV-23 and newer PCVs. Unlike the plain polysaccharide vaccines, the protein-conjugated PCVs are T-cell dependent and capable of inducing immunity in infants, resulting in high-affinity antibodies with isotype switching, affinity maturation and generation of immunological memory. The PCVs vary in their number of pneumococcal serotypes (valency) and the conjugate proteins used. The 7- (PCV-7) and 13-valent PCV (PCV-13) are conjugated to the non-toxic *C. diphtheria* CRM_197_ protein. In many affluent countries, PCV-13 has replaced PCV-7, while other countries use the 10-valent pneumococcal-*Haemophilus influenzae* Protein D conjugate vaccine (PHiD-CV). The PHiD-CV vaccine utilises *H. influenzae* protein D (PD) (for 8 serotypes), tetanus (serotype 18C) and diphtheria toxoid (19F) as its conjugating proteins (26, 27). PD is a highly-conserved outer membrane lipoprotein, which is immunogenic in both encapsulated (typeable) and non-typable *H. influenzae* (NTHi) species (28), although evidence of it inducing protection against these pathogens remains limited (29, 30).

All PCVs are highly effective and provide almost complete protection against invasive pneumococcal disease from vaccine-type serotypes (26). For our randomised controlled trial (RCT), we chose PHiD-CV instead of other PCVs for several important reasons. Firstly, in the same target population as our RCT, a 3-arm RCT (*n* = 227) demonstrated that PPV-23 given to pregnant First Nations women (compared with controls) did not reduce ear disease or nasopharyngeal pneumococcal carriage (31). Likewise, seven RCTs in other population groups using PPV-23 in the third trimester did not reduce pneumococcal infections and/or nasopharyngeal (i.e., upper airways) carriage in infants (14, 31). The non-efficacy of PPV-23 is not surprising as it is an inferior vaccine to PCVs being T-cell independent and thus inducing primarily IgM responses with little isotype switching to IgG or IgA antibodies and is unable to initiate immunological memory (14), which is consistent with clinical findings (23).

Secondly, PCVs that use CRM_197_ as the conjugating protein have been associated with hyporesponsiveness or immune tolerance to vaccine antigens (32, 33). The sole maternal immunisation RCT using a PCV found that infants of mothers randomised to a 9-valent prototype PCV had significantly reduced antibody responses to six PCV serotypes and increased ear disease at 6-months (32). As the conjugate protein for PCV-9 is the same as PCV-7 and PCV-13 [but different to PHiD-CV (26)], using PCV-13 may lead to comparable adverse events. Furthermore, hyporesponsiveness to PCV-7 was found in infants with prior natural infections and carriage of vaccine type strains (33). As First Nations infants have early onset and very high rates of pneumococcal carriage and ear infections (34), using PCV-13 for maternal immunisation may further increase the risk of these adverse events.

Thirdly, compared to other PCVs, PHiD-CV may provide additional *H. influenzae* protection for preventing infant ALRI (28). Mice immunised with PD (conjugating protein in PHiD-CV) had significant protection against *H. influenzae* lung infection and otitis media (28). *H. influenzae* are important pathogens in child and adult ALRIs (35, 36) and chronic lung diseases (37, 38). A systematic review (36) and a study employing transthoracic needle lung aspirations (35) described *H. influenzae* (mostly NTHi) as being second only to pneumococci as the most common cause of pneumonia in children from LMICs.

Fourthly, although maternal PHiD-CV might therefore be more beneficial and cost-effective (24) than PCV-13 at reducing infant ALRIs, there are currently no maternal immunisation studies using PHiD-CV.

In addition to the above, in First Nations children living in the Northern Territory of Australia, *H. influenzae* colonisation occurs early in infancy (34) and is an important cause of lower airway infections (35, 37). Further evidence of *H. influenzae* role in lower airway infections in children are: (a) *H. influenzae* is prevalent (30–85%) in the lower airways of children with bronchiectasis (38); (b) *H. influenzae* infection (most found to be were NTHi) in children with protracted bacteria bronchitis [a precursor of bronchiectasis (39, 40)] conferred >7-times higher risk of future bronchiectasis at the 2 year follow-up [hazard ratio (HR) = 7.6, 95% CI 1.7, 34.3] compared when *H. influenzae* was absent (41) and at the 5 year follow-up (Odds Ratio_adjusted_ = 5.1, 95%CI 1.4–19.1) (42); (c) PHiD-CV vaccination improved NTHi-specific cell- and antibody-mediated immune responses in children with bronchiectasis (25) and; (d) vaccination with PHiD-CV was associated with a significant reduction of NTHi infection in the lower airways of children (43).

In the context of the above, our RCT's primary aim is to determine whether vaccinating pregnant women with PHiD-CV reduces ALRIs in their infants in the first year of life. Our primary hypothesis is that maternal PHiD-CV immunisation will reduce the incidence of ALRI episodes in their infant's first year of life, compared to those who did not receive the vaccine.

Our secondary aims are to evaluate the impact of maternal vaccination with PHiD-CV upon:

- Time-to the-first infant ALRI event using different ALRI definitions;
- Adverse events;
- Nasopharyngeal carriage of pneumococcal vaccine-type serotypes and *H. influenzae* at birth, 6 and 12-months of age; and
- Immune responses [vaccine-type antibody responses (total IgG to both vaccine-type pneumococcal serotypes and PD) and cell-mediated (cytokines) immune responses to pneumococci, NTHi and PD] in a subset of infants.

## Methods and Analysis

### Design

We are undertaking a parallel, single-blinded multicentre RCT (with concealed 1:1 allocation) to determine the efficacy of maternal immunisation with PHiD-CV at reducing ALRIs in infants during their first year of life. We randomised the first participant on 14th January 2019 and we anticipate continuing recruitment until late 2023. We currently have four study centres and six recruiting hospital sites: one centre in Malaysia (Likas Hospital in Kota Kinabalu) and three in Australia [Darwin (Royal Darwin Hospital, Darwin Private Hospital and Palmerston Regional Hospital) in the Northern Territory; and Brisbane (Royal Brisbane and Women's Hospital, RBWH), and Logan city (Logan Hospital) in South-East Queensland]. Previously we had a fifth study centre in Toowoomba (regional Queensland) whereby a research nurse was stationed at an Aboriginal Medical service, but after a year of unsuccessful recruitment, we abandoned this centre. Pending recruitment numbers and remaining funds, other centres/hospital sites may be included in the future.

### Study Population

#### Inclusion Criteria

(i) Generally healthy women aged 17–40 years; (ii) singleton pregnancy between 27^+6^ and 34^+6^ weeks gestation at the time of randomisation, (iii) planned delivery at study hospitals with delivery suites (Likas Hospital, Royal Darwin Hospital, Darwin Private Hospital, RBWH and Logan Hospital), (iv) the infant will be identified as either First Nations peoples (Australian Aboriginal and Torres Strait Islanders or Maori and Pacific Islanders from migrant families to Australia) or Malaysian, (v) treating obstetrician/medical practitioner managing the pregnancy approves the participant's enrolment in the study, (vi) provision of written informed consent and willing and able to meet the protocol requirements, and (vii) not planning to move from the study area before the infant reaches 12-months of age.

#### Exclusion Criteria

(i) Contraindication or known sensitivity to PHiD-CV, (ii) immune deficiency or suppression (primary or secondary); (iii) receipt of any pneumococcal vaccine in the previous 2-years; (iv) history of severe allergy e.g., anaphylaxis; (v) previously enrolled or (vi) pre-existing illness considered as a high-risk pregnancy (Table 1), (vi) current (or within 90-days prior to receiving study vaccine) or planned (during the active study period) immunosuppressive therapy, including systemic corticosteroids (≥14-days exposure in a 30-day period); (vii) administration of immunoglobulins and/or blood products, with the exception of Rh Immune Globulin, within 90-days prior to receiving the study vaccine, or planned administration of such products during the study period; (viii) active participation in a clinical trial of another investigational drug/vaccine or interventional therapy, or (ix) other conditions that the investigators/treating physicians consider should be excluded from the trial to prevent potential harm/risk to the subject or may adversely affect study outcomes.

**Table 1:** Definition of high-risk pregnancies.

**Maternal illnesses**
•Diabetes mellitus diagnosed prior to pregnancy OR if diagnosed during pregnancy the treating obstetrician considers the person's glycaemic control status warrants exclusion from the study.
•Hypertension (resting blood pressure ≥140/90) requiring antihypertensive therapy
•Heart disease diagnosed by a cardiologist
•Active and/or unstable autoimmune disease on medication
•Confirmed and documented chronic renal failure
•Serious neurological disease (including epilepsy requiring drug therapy)
•Psychiatric disorder on medication known to be teratogenic (other psychiatric conditions are discussed on a case-by-case basis with local obstetrician and central office^*^).
•Cirrhosis
•Deep vein thrombus (current or recent in pregnancy)
•Uncontrolled thyroid disease (subclinical hypothyroidism may be included)
•Pulmonary disease that is not controlled by medications and/or FEV_1_ <70% using ethnic corrected values
•Substance abuse – judged on a case-by-case basis and in consideration of what substance is involved and how this might impact upon study participation (including alcohol and tobacco use)
**Pregnancy-related conditions**
•Malaria during pregnancy
•RBC Isoimmunisation
•≥3 spontaneous abortions AND not given birth to a healthy infant previously
•Previous stillbirth or neonatal death AND not given birth to a healthy infant previously
•Previous infant with known genetic disorder (discussed on a case-by-case basis with central office^*^)
•Previous infant with a major congenital anomaly (discussed on a case-by-case basis with central office^*^)
•Multiple pregnancy
•Expected to give birth to an infant with a major congenital abnormality
•Delivery <37-weeks expected or highly likely - (discussed on a case-by-case basis with central office^*^). Examples may include short cervix, incompetent cervix, past-history of spontaneous pre-term birth.
•Polyhydramnios (defined as either a deep vertical pocket >8cm or an amniotic fluid index >24cm on ultrasound examination) or oligohydramnios (defined as a maximum vertical pocket <2cm or amniotic fluid index <5cm on ultrasound examination)
•Intrauterine growth restriction or foetal growth restriction (<10th percentile) or macrosomia (>97th percentile for ethnicity)
•Preterm rupture of the membranes has occurred by the time of screening/enrolment
•Pre-eclampsia

* *Central office refers to the lead investigator (ABC)*.

### Study Protocol

**Figure 1:**
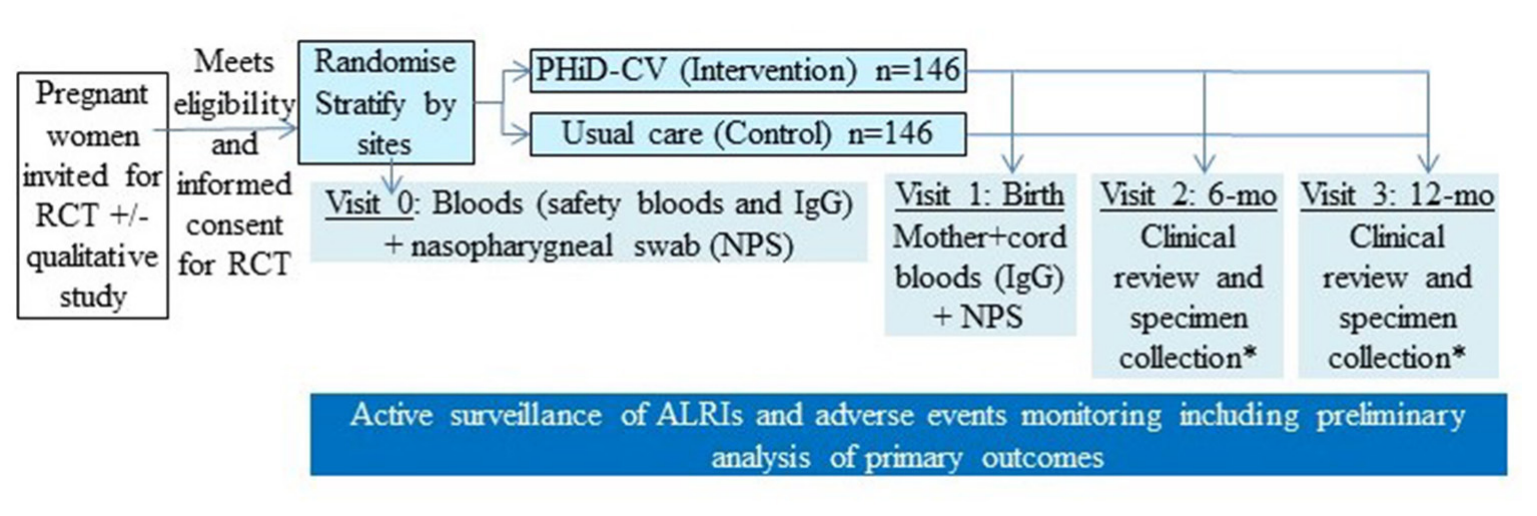
Overall schematic study design of our randomised controlled trial (RCT). *In a subset of infants, specimen collection includes nasopharyngeal swab (NPS) and infant blood (for immunoglobulins and peripheral blood mononuclear cells). PHiD-CV, 10-valent pneumococcal conjugate *Haemophilus influenzae* (Hi) Protein D vaccine.

Pregnant women potentially eligible for the study are approached during a routine antenatal visit. Information is provided and on follow-up, they are invited to participate in the RCT. After confirming eligibility and providing informed consent, enrolled pregnant women are randomised to one of the two arms (intervention or control), as summarised in the Figure 1.

#### Intervention and Control

Women randomised to the intervention arm receive a single dose of PHiD-CV (Synflorix®, GSK Biologicals, Belgium) between 27^+6^ and 34^+6^ weeks gestation. Those randomised to the control arm receive usual care (no PHiD-CV). If not already administered, seasonal influenza and dTPa vaccines are also offered at the same time to all women in both study arms as part of standard care.

#### Randomisation, Allocation Concealment and Blinding

At enrolment, participants are randomised to one of the two study arms (intervention or control). The random treatment allocation is computer-generated, stratified by site, using permuted blocks sizes of various sizes, concealed and supervised by a statistician.

Participants and study teams are unaware of the treatment to be allocated before the patients are entered into the study. The study is not placebo-controlled, but assessors collecting study outcome data are blinded to treatment allocation.

#### Data Collection, Monitoring and Follow-Up Details

All data collected are being recorded by good clinical practise trained nurse researchers on standardised data sheets as performed previously in our many RCTs (44–46). Most women and their infants are followed in their local community/region where the infants also receive their routine vaccinations following the national infant immunisation schedules for either Australia or Malaysia (Table 2). Immunisations are confirmed by checking the Australian Immunisation Register and the infants' individual paper-based health records in Malaysia.

**Table 2:** Infant immunisation schedules for the Northern Territory and Queensland, Australia and Sabah, Malaysia.

**Age**	**First Nations infants, Australia**	Other Australian infants^*^	**Sabah, Malaysia**
Birth	HB	HB	HB
			BCG
2-months	DTaP-IP-HB-Hib	DTaP-IP-HB-Hib	DTaP-IP-HB-Hib
	PCV-13	PCV-13	PCV-13
	Meningococcal B	Rotavirus	
	Rotavirus		
3-months			DTaP-IP-HB-Hib
4-months	DTaP-IP-HB-Hib	DTaP-IP-HB-Hib	PCV-13
	PCV-13	PCV-13	
	Meningococcal B	Rotavirus	
	Rotavirus		
5-months			DTaP-IP-HB-Hib
6-months	DTaP-IP-HB-Hib	DTaP-IP-HB-Hib	Measles
	PCV-13		PCV-13
9-months			MMR
12-months	PCV-13	PCV-13	MMR
	Meningococcal B	Meningococci A,C,W,Y	
	Meningococci A,C,W,Y	MMR	
	MMR		

* *Maori and Pacific Island infants*.

*HB, hepatitis B; BCG, Bacille Calmette-Guerin; DTaP-IPV-HB-Hib, diphtheria-tetanus-acellular pertussis-inactivated poliomyelitis vaccine-hepatitis b-Haemophilus influenzae type b; PCV-13, 13 valent pneumococcal conjugate vaccine*.

At enrolment, maternal baseline specimens [blood, nasopharyngeal swabs (NPS)] and demographic/clinical data are being collected (e.g., age, gender, family and household size, tobacco smoking, family and immunisation history, medications, co-morbidity) from the woman and their medical notes. Participants are contacted at least monthly using a variety of communication tools [telephone, short messaging service (SMS) texting, emails and/or home visits].

Women are seen at delivery and specimens (blood, NPS) collected. Infants are followed for 12-months with specimens collected at birth (cord blood, NPS). The infants are also seen with additional specimens collected (when possible) at ages 6-months (clinical review, blood, NPS) and 12-months (clinical review, blood, NPS).

Infants' data are obtained from parents/carers and the medical notes at every visit (birth details, breast feeding, growth, respiratory symptoms/signs and ALRIs co-morbidities). We have established robust methods for capturing ALRI episodes from our previous RCTs (44, 45, 47). Hospital records will be reviewed for identifying hospitalisations. We are also contacting the infants' primary care physician for verification of ALRIs. Adverse events (see below) are recorded following the current guideline definitions (19).

*Exit criteria* are: consent withdrawn, infant born with congenital disorders affecting ALRI frequency (e.g., chromosomal abnormalities, genetic syndromes, primary immuno-deficiency, airway, cardiac or neuromuscular disorders), or death.

### Laboratory-Based Methods

#### NPS Bacteriology

NPS are stored and processed as described previously (38, 44, 48). Batches of swabs will be thawed and cultured on selective media. Growth of pneumococci and *H. influenzae*, including NTHi, will be confirmed by standard techniques (43, 44). *H. influenzae* will be differentiated from *H. haemolyticus* isolates by targeting the *siaT* gene using real-time polymerase chain reaction assays (49). Pneumococcal serotypes will be determined later by Quellung serotyping methods (50).

#### Peripheral Blood Mononuclear Cell (PBMC), NTHi and Pneumococcal Driven Systemic Immune Responses

In all, 2–3 mls of blood collected are processed and cryopreserved as described previously (25, 51) using standardised methods. We have established previously the preparation of the NTHi inoculum, cell culture and immunoassays (25, 51). Briefly, PBMC (1 × 10^6^ cells/ml) will be challenged with live NTHi or pneumococci (4 × 10^6^ colony forming units/ml), phyto-hemagglutinin (PHA; positive control) or medium alone (baseline control). Stored bronchoalveolar lavage (BAL) isolates (48) that are proven viable will be used. We will use two non-vaccine pneumococcal serotypes (15A, 15B) and two vaccine-type serotypes (19F, 23F), the commonest serotypes found in our BAL studies (48).

Cytokines, representative of the innate (TNF-α), Th1 (IFN-γ) and Th2 (IL-6, IL-13) immune responses and regulation of inflammation (IL-1β,) will be measured in culture supernatants at 24 or 72-h, as optimised previously, using a Dissociation-Enhanced Lanthanide Fluorescence Immunoassay (DELFIA™) (51, 52). Additional cytokines may be performed pending new data at the point when the specimens are analysed at the end of the study. DELFIA™ has a large dynamic range (~ 3–30,000 pg/ml) with low background interference. All samples for immunology-related work will be batch-tested to minimise inter-assay variation.

#### Serum IgG Measurements

Total serum IgG antibodies specific for PD and selected pneumococcal serotypes (both vaccine and non-vaccine types, yet to be determined) will be undertaken using methods described previously in our studies (25, 31).

### Outcomes

All clinic staff (other than the nurse administering the study vaccine) and persons collecting the outcome data are blinded to the mothers' allocation group. Our main outcomes are at 12-months as ALRIs early in life increase the risk for subsequent hospitalisations for ALRIs later in infancy (53–55) and it is a more robust outcome for comparative epidemiological studies.

Our case definition of ALRI is a medically-attended episode of illness defined as the presence of acute cough, dyspnoea, raised respiratory rate for age, new abnormal chest examination findings or abnormal radiographic findings for presumed respiratory illness (clinic visits for ALRIs within 2-weeks of each other will be counted as part of the same ALRI episode).

*The primary outcome* is the rate of medically-attended ALRIs at age 12-months of our endpoint case definition that fulfils any of the below, i.e., a composite single outcome:

- Case definition confirmed via medical records;
- Case definition suspected via medical records (i.e., some information in the above is missing and the case definition above cannot be confirmed); or
- Case definition based upon parent reported ALRI: parent reports a medically-attended episode of a respiratory illness for which antibiotics were prescribed.

We also plan a sensitivity analyses based on episodes meeting the criteria of

- (i–iii) with antibiotics prescribed.
- (i) and (ii) only.
- (i) only.

#### Secondary Clinical Outcomes

1. Pneumonia confirmed by chest radiograph with: (a) antibiotics given (b) no antibiotics given;
2. Clinical pneumonia defined as cough or difficulty breathing for <14-days accompanied by: raised respiratory for age (>60 breaths per minute if aged 0 to <3-months, ≥50 breaths per minute for those aged 3 to 12-months), lower chest wall indrawing, nasal flaring, grunting, dull chest percussion note, coarse crackles, or bronchial breathing, peripheral oxygen saturation <92%, altered consciousness, inability to sit or feed, convulsions (56) with: (a) antibiotics given (b) no antibiotics given;
3. Hospitalised ALRI episodes with: (a) antibiotics given (b) no antibiotics given;
4. Time-to-first ALRI episode (defined as medically-attended ALRI with antibiotics prescribed) and respiratory-related hospitalisation;
5. Proportion of children with any medically-attended ALRI;
6. Adverse events related to vaccination, pregnancy and infants (as separate outcomes) using definitions outlined by the GAIA series of papers (19).

#### Secondary Lab Outcomes in a Subgroup of Infants (Estimated *n* = 70)

- Vaccine-type pneumococcal IgGs [(maternal: baseline, birth); (infant: cord, 6-months and 12-months of age)];
- Cytokines from the infant's systemic immune responses *ex-vivo* (TNF-α, IFN-γ, IL-6, IL-13, IL-1β) from PBMCs stimulated with NTHi (51) and pneumococci (at least one each of vaccine and non-vaccine types); and
- Nasopharyngeal carriage of NTHi and pneumococci [(maternal: baseline, birth); (infant: birth, 6-months and 12-months of age)].

#### Halting Rules

The study is being monitored by an external independent data safety and monitoring committee (iDMC) with predefined halting rules for harm. This includes increased adverse events in the mother, foetus or infants, or if the difference between groups at the interim analysis (when 50% of the sample size participants have each provided at least 6-months of data) reaches a *p* ≤ 0.01.

#### Data Monitoring, Management and Analyses

All adverse events, including serious adverse events, are monitored by an iDMC, which was established and met prior to commencing the study. A First Nations Reference Group based at the Menzies School of Health Research in Darwin oversees the cultural aspects of the study. Data coding and entry is coordinated in Brisbane and conducted in accordance with good clinical practise.

The analyses will be overseen by a biostatistician. Results will be reported and presented in accordance with CONSORT guidelines. We will use the “intention-to-treat” approach for the main analyses. We also plan a “per-protocol” analysis. A detailed statistical analysis plan will be developed prior to undertaking the final analysis. Any exploratory, *post-hoc* or unplanned analysis will be identified clearly.

### Primary Outcome

The main effects of the intervention will be determined by comparing the primary outcome of medically-attended ALRI rates in the first year of life recorded between the two groups (PHiD-CV vs. controls). We will report the incidence rate ratio (IRR) using negative binomial regression as well as the absolute difference between groups (with corresponding 95% CIs).

Sensitivity analyses for the primary outcome will be performed based upon the ALRI classification (described above). Also, we plan a preliminary analysis when the primary outcome for 50% of the sample size participants is available and each provides at least 6-months of individual data. We plan using 6-months of data (rather than 12-months, which is the time point for our RCT's outcome) for safety reasons, based on adverse events found in the prototype PCV-9 RCT (32).

### For Secondary Aims

We will compare the inter-group proportion of children with any hospitalised ALRI, clinical pneumonia and radiographically-confirmed pneumonia using ORs with logistic regression (and 95%CIs). In addition, we will construct a Kaplan-Meier curve for each group for time-to-first ALRI episode and respiratory-related hospitalisation, perform a log-rank test and report a HR (using the Cox regression model).

For the secondary laboratory-based aims, we will compare between-group differences for: (i) IgG geometric means against PD (25) and serotype-specific pneumococci (31) using Student's T-test after logarithmic transforming the data (+/- maternal pre-vaccine levels adjustment); (ii) cytokine levels expressed by PBMCs after stimulation by live NTHi and pneumococci [Mann-Whitney (25, 51)]; and (iii) proportions of nasopharyngeal cultures positive for *H. influenzae* and pneumococci (vaccine and non-vaccine types) using Chi^2^ tests and reporting ORs (95%CIs) (44, 45).

The proportion of participants (pregnant participant and infants) with any adverse events will be compared between groups, using Chi^2^ tests and reporting ORs (95%CIs) (44, 45).

#### Sample Size

Based upon our primary outcome (medically-attended ALRI rate), we wish to detect a significant difference between infants born to mothers (a) immunised (active), and (b) not immunised (controls), with PHiD-CV. A total sample size of 266 infants with complete data at 12-months of age (133 per group) will provide 88% power to detect a 30% decrease (IRR = 0.7) in the rate of ALRIs per infant-year from 3 ALRIs per infant-year in the control group to 2.1 ALRIs per infant-year in the intervention group, assuming a shape parameter of 0.46 reported by Keene et al. (57). To account for 10% attrition, we will recruit a total of 292 participants (146 per group).

We assumed a 30% reduction in the ALRI rate based upon the effect of PHiD-CV on BAL-defined NTHi lower airway infection in our previous work (43) and published data of vaccine efficacy of 26–29% in hospital-diagnosed community acquired pneumonia in two different (3 + 1 or 2 + 1) infant vaccination schedules utilised in the FinIP study (58). We conservatively assumed a medically-attended ALRI rate of 3 per year in the controls based upon a previous study from Northern Territory remote communities (59). In our cohort study of First Nations infants based at a Caboolture (outer metropolitan Brisbane) Aboriginal Medical Centre, the medically-attended ALRI rate was also 3 per year (60). We assumed a low drop-out rate since we expect to collect our primary outcome data from almost all children using electronic medical records as done previously (44).

#### Impact of Severe Acute Respiratory Syndrome Coronavirus 2 (SARs-COV-2) Upon the Study

The current pandemic has substantially affected many aspects of our RCT. Firstly, recruitment was severely impacted by strict state and regional lockdowns with travel restrictions preventing face-to-face visits required by our study. Secondly, trust in vaccinations was also adversely affected by mixed health messaging and controversy theories fuelled by social media. Thirdly, the incidence of medically-attended ALRIs is substantially lower than that predicted, a phenomenon found worldwide coincident with the public health measures enacted to reduce the spread of SARs-COV-2 (61). Fourthly, there are also financial consequences as no additional funds were made available by the funding body [Australian National Health and Medical Research Council (NHMRC)] and research team members continued to be paid throughout this pandemic. Overall, we anticipate that our study's findings will be substantially affected by the ongoing current pandemic.

## Discussion

The global burden of ALRIs has reduced since the turn of the twenty first century, but remains high (1). ALRIs are more common in some settings than others, such as in LMICs and among First Nations children living in high-income countries (1, 3). This high burden has public health implications, not only because of its associated mortality but also its morbidity in young children, which is linked to future impaired respiratory health and lung function deficits (4, 6). Novel strategies are required to reduce the burden from ALRIs in young children. One such strategy is maternal immunisation (14). Currently, we are undertaking a multicentre, assessor-blind RCT across two countries to address the question of whether vaccinating pregnant First Nations and Malaysian women with single dose PHiD-CV (compared to controls) reduces ALRIs in their infants in the first year of life.

Our target group is a population at increased risk of ALRIs. Within days of their birth, Australian Northern Territory First Nations infants are colonised by potential respiratory bacterial pathogens (pneumococci, *H. influenzae* and *Moraxella catarrhalis*) in their nasopharynx (34). This colonisation occurs at a much younger age and at a higher density than in other Northern Territory infants (34). Moreover, in these young infants bacteria can reach the lower airways by recurrent episodes of micro-aspiration (62), especially when nasopharyngeal secretions are copious and dense with bacteria (38). Our BAL cultures from the lower airways of young First Nations children with bronchiectasis also showed greater bacterial density and diversity than found in non-first Nations children with protracted bacterial bronchitis (38). Furthermore, most pneumococcal serotypes colonising the nasopharynx can cause lower airway infection (48). Even if initially a viral infection, secondary bacterial infection may ensue as bacterial densities increase further within the upper airways (63), especially in high-risk, heavily colonised. First Nations children (64) and those with a history of recurrent ALRIs. These events can occur in the first months of life before routine childhood vaccines are administered and provide protection.

Our RCT targeted First Nations peoples because of their known increased risk of ALRIs and their high prevalence of chronic lung disease in children (3, 7, 8). We also included Malaysian women for several reasons. Firstly, this will improve generalisability of our RCT by including a LMIC study centre. Secondly, at this site, there is relatively high mortality and morbidity from ALRIs, including pneumonia (65), and chronic suppurative lung disease is a recognised health problem (66). Finally, the senior researchers have established successful collaborations (47).

While First Nation children's immunisation coverage is generally high (>90%), it is often delayed (67). During this vulnerable period, robust immune responses are required to combat ALRIs. Vaccinating pregnant women provides infants with maternally-derived antibodies during the first 4-6 months of life (14, 16). This is a period of rapid lung growth where infectious insults may have long-term consequences by interrupting lung development and possibly also altering immune programming (68). Thus, host defences boosted by vaccine-induced maternal antibodies could protect the infant from early ALRIs and their late sequelae.

Preventing infant ALRI could also reduce subsequent hospitalisation. An Australian study found that ALRI in early infancy significantly increased the risk (HR = 3, 95%CI 2.6–3.4) for further respiratory hospitalisation after adjusting for risk factors (smoking, gestational age, season, delivery mode), with a dose-response effect (54).

There are eight published maternal immunisation studies using PPV-23 in the third trimester. None have shown any clinical benefit for respiratory infections (18, 31). The newer PCVs are more immunogenic and effective than the 3-decade old PPV-23 (14, 23). Our reasons for choosing PHiD-CV, instead of other types of PCVs, were provided in the Introduction. Furthermore, we are using a single PHiD-CV dose (rather than multiple doses) for our RCT as a single dose of PCV induces robust T-cell responses and B-cell memory in adults (23) and older children (27). Studies show that antibody responses against vaccine serotypes are above the WHO-defined immune correlate of protection level threshold of 0.35 ug/ml (27) and current guidelines recommend single PCV doses in adults (18, 20, 23). This is in contrast to the multiple doses required for priming infants (27) who have immature immunity.

Published maternal immunisation studies using inactive (as opposed to live) vaccines (PHiD-CV is an inactive vaccine) have shown excellent safety and cost-effectiveness when efficacious (14, 18). Longitudinal and surveillance studies have not detected increased risks of adverse pregnancy events or congenital defects (14, 69). A recent RCT involving pregnant Brazilian women with human immunodeficiency virus infection reported PHiD-CV was a safe vaccine (70). The Brazilian study also described that both PHiD-CV and PPV-23 conferred similar levels of seroprotection to infants when measured at 8-weeks of age (70). However, unlike administering PCVs during the neonatal period, maternal immunisation may interfere with subsequent vaccine responses in infants (71). Indeed, one RCT using PCV-9 reported negative outcomes in infants at 6-months, but not at 12-months of age (32). Nevertheless, caution is needed when interpreting these data (32) as a family history of grommets was more strongly associated with ear disease in the vaccine group; thus blinding may have been incomplete. The authors speculated that the negative outcome in this study related to altered B-cell memory responsiveness in infants following exposure to certain antigens while *in-utero* resulting in attenuated immune responses to conjugated vaccines (32, 71). For this reason, our RCT will incorporate a preliminary safety analysis when 50% of the infant participants have reached 6-months of age.

In a subgroup of infants, we are undertaking immunological investigations. We wish to identify if immunisation increases maternal and cord blood IgG antibodies to vaccine-type pneumococcal serotypes and to *H. influenzae* PD. This is significant as a recent study from Papua New Guinea suggested that antibodies to NTHi outer membrane proteins may not be transferred from mothers to their infants (72). We will also determine innate and systemic immune responses to pneumococci and *H. influenzae* in both mothers and their infants. Our earlier studies demonstrated differences in innate and systemic immune responses between 80 children with bronchiectasis and 51 healthy controls (51). Compared with healthy controls, PBMCs isolated from children with bronchiectasis had deficient interferon (IFN)-γ response *ex-vivo* when challenged with live NTHi isolates (51). In contrast, systemic cellular responses to non-specific mitogens remained similar. We then found that vaccination with PHiD-CV improved NTHi-specific cell- and antibody-mediated immune responses that approached levels achieved by healthy control children (25). Thus, in our RCT we will examine these immune responses using NTHi clinical isolates and the most common pneumococcal serotypes detected in BAL cultures (48). Such a study has never been undertaken within a RCT.

First Nations children and children from LMIC are heavily colonised in their nasopharynx by respiratory bacterial pathogens from a very young age (34) and to date PCVs have had limited or no impact upon either pneumococcal or *H. influenzae* colonisation and density or pneumococcal serotype diversity (73, 74). However, some experts argue that provided vaccines still protect against disease, this lack of effect upon colonisation may not be a disadvantage ecologically if it minimises strain replacement (75). Our study will assess the combined impacts of maternal and infant PCV immunisation upon pneumococcal and *H. influenzae* colonisation of the nasopharynx.

While our study has many novel and robust aspects, there are also limitations. Ideally, our Australian study centres and sites would have included communities most at risk of ALRIs as in Central Australia. However, we were unable to obtain ethics clearance from the responsible committee (based in Adelaide). This was despite receiving support from the First Nations community leaders and elders and the relevant local Aboriginal controlled medical service, the safety profile of maternal vaccines and potential for large clinical benefits mentioned above. In addition, we elected to use a range of definitions for ALRI episodes in our RCT, in light of the current difficulties and controversies over defining ALRIs in the community including pneumonia without a chest radiograph in clinical studies (6, 76–78). Finally, funding restrictions preclude us from collecting a NPS from infants at 4-weeks of age to help identify whether maternal PHiD-CV delays early pneumococci and *H. influenzae* colonisation.

In this protocol paper, we have described our RCT and the main immunology outcomes related to maternal PHiD-CV immunisation in the third trimester of pregnancy. We also plan additional side studies that have not been included in this protocol paper. Our RCT has the potential to prevent early and recurrent ALRIs and thus preserve lung health during the vulnerable period of maximum post-natal lung growth (79). Our study addresses a large knowledge gap concerning a common worldwide problem in early childhood and if found to reduce ALRIs it will have a global impact. The multicentre nature of our study increases the generalisability of the future findings of our RCT, which is also complemented by assessing immunological outcome in a subset of participants. If successful, our RCT is likely to influence national and international immunisation policies.

## Ethics Statement

The Human Research Ethics Committees (HRECs) of all the recruiting institutions and the academic institutions. These are: Metropolitan South Hospital and Health Service 2018/QMS/44099, Queensland University of Technology HREC 180000084 and The University of Queensland HREC 2019000053 for Royal Brisbane & Women's Hospital and Logan Hospital sites, Northern Territory Department of Health and Menzies School of Health Research 2018-3128 for the Darwin sites, and Malaysia Medical Research and Ethics Committee 18-3427-43934 for the Malaysian site at Likas Hospital. All have approved the research protocol version 7. Written informed consent to participate in this study was provided by the participants' legal guardian/next of kin.

## Author Contributions

ABC conceived and designed the study, drafted the manuscript, and was primarily responsible for obtaining the grant. KG had a major input in editing the manuscript. ABC, KG, MB, MT, HS-V, PT, HM, GS, and SP are chief investigators in the NHMRC grant and contributed to study design and manuscript. MC (biostatistician) and PM (clinical triallist) also contributed to the study design. SF is the lead investigator in Kota Kinabalu, KL is the lead investigator at the RBWH, DS and HU are lead investigators at the Darwin site, and HE and JO are lead investigators at Logan. RM is currently coordinating all aspects of the project. SY and HO'F are coordinating the biological specimens. JM and CM are medical assessors. All authors read and approved the final manuscript.

## Funding

This study was funded by a 5-year Australian NHMRC project grant (Number 1138555) and supported by a NHMRC Centre for Research Excellence in Bronchiectasis for Children (Grant Number 1170958; www.crelungs.org.au). ABC is supported by a NHMRC senior practitioner fellowship (Grant 1154302). HM is supported by a NHMRC practitioner fellowship (Grant 1155066).

## Author Disclaimer

The views expressed in this publication are those of the authors and do not reflect the views of the NHMRC.

## Conflict of Interest

KG participated in a rotavirus strain outbreak advisory board for GlaxoSmithKline, Rixensart, Belgium. ABC has received fees to the institution from work relating to IDMC membership of an unlicensed vaccine (GSK), and a COVID-19 vaccine (Moderna) outside the submitted work. The remaining authors declare that the research was conducted in the absence of any commercial or financial relationships that could be construed as a potential conflict of interest.

## Publisher's Note

All claims expressed in this article are solely those of the authors and do not necessarily represent those of their affiliated organizations, or those of the publisher, the editors and the reviewers. Any product that may be evaluated in this article, or claim that may be made by its manufacturer, is not guaranteed or endorsed by the publisher.
